# Successful Treatment of Refractory Mucocutaneous Behçet's Disease With Gastrointestinal Involvement Using Anti-tumor Necrosis Factor-Alpha (Anti-TNF-α) Therapy

**DOI:** 10.7759/cureus.84937

**Published:** 2025-05-27

**Authors:** Manar Elfatih Mohamed, Raghad Mazin Al-Issa, Hassan Ahmed, Ali Emad Al-Zaidy, Faisal Elbadawi

**Affiliations:** 1 Internal Medicine, Postgraduate Medical Education Division, Mohammed Bin Rashid University, Dubai, ARE; 2 Rheumatology, Postgraduate Medical Education Division, Mohammed Bin Rashid University, Dubai, ARE; 3 Gastroenterology, Postgraduate Medical Education Division, Mohammed Bin Rashid University, Dubai, ARE; 4 Rheumatology, Dubai Health, Dubai, ARE

**Keywords:** anti-tnf-α treatment, behcet’s disease (bd), disease-modifying anti-rheumatic drugs (dmard), gastrointestinal behcet’s disease (gibd), mucocutaneous manifestations

## Abstract

Behçet's disease is a systemic inflammatory disorder that manifests with recurrent oral and genital ulcers, skin lesions, and ocular disease. Current available classification criteria greatly depend on mucocutaneous manifestations. Gastrointestinal involvement is particularly rare and often presents significant diagnostic and therapeutic challenges. We report the case of a 33-year-old male with uncontrolled Behçet’s disease, presenting with cutaneous vasculopathic ulcers and gastrointestinal manifestations despite being on conventional disease-modifying anti-rheumatic drugs (CDMARDs), who achieved full remission following initiation of adalimumab biosimilar. This case highlights the effectiveness of adalimumab biosimilar in managing refractory mucocutaneous and gastrointestinal manifestations of Behçet’s disease. Most of the evidence available is from observational data. There is a growing body of evidence supporting the use of anti-tumor necrosis factor-alpha (TNF-α) therapy in severe gastrointestinal Behcet’s disease. Anti-TNF-α may be considered in some cases.

## Introduction

Behçet’s disease (BD) is a chronic vasculitis affecting blood vessels of variable sizes, both venous and arterial circulations. It is more commonly found along the historic Silk Road (from East Asia to Mediterranean countries) [1]. It is a multisystemic disease with a relapsing and remitting course, mainly involving skin and mucosa; however, it can affect joints, eyes, blood vessels, and gastrointestinal (GI) and neurological systems, with the latter four being associated with a poorer prognosis, especially if left untreated [2]. It has been proposed that BD is an autoimmune and inflammatory process that is triggered by infection or environmental factors in genetically susceptible people [2]. There is no specific test for BD, and the diagnosis mainly depends on the overall clinical picture using international criteria aligned with ruling out other differential diagnoses. 

Mucocutaneous manifestations of BD include oral and genital ulcers (typical presentation), erythema nodosum, papulopustular lesions, and pyoderma gangrenosum. Colchicine is being used as a first-line treatment to prevent recurrent mucocutaneous lesions. If colchicine fails, other treatment strategies include immunosuppressive or biologic agents such as azathioprine or anti-tumor necrosis factor-alpha (TNF-α). Topical steroids can be applied to promote healing in acute exacerbations of mucocutaneous manifestations. Leg ulcers could occur due to venous circulatory stasis or obliterative vasculitis, and management should be multidisciplinary, including a dermatologist and vascular surgeon [3]. It may coexist with pyoderma gangrenosum in some cases, warranting immunosuppressive treatment.

Gastrointestinal involvement of Behçet’s disease (GIBD) presentation is nonspecific, including symptoms of abdominal pain, diarrhea, and gastrointestinal bleeding. It is characterized by the presence of ulcers in the terminal ileum and cecum in 70% of cases in one study, although it can involve any part of the gastrointestinal tract [4]. Hence, imaging and/or endoscopy are required to rule out other differentials that have similar presentations, including inflammatory bowel disease and infections such as tuberculosis. 

The treatment guideline for GIBD is mainly based on retrospective observational studies, as there are no clinical trials considering the rarity of this system's involvement. The management depends on the severity of the disease, with glucocorticoids being used in acute exacerbation. 5-aminosalicylate derivatives can be used in milder cases, whereas immunosuppressive agents like azathioprine and biologics like infliximab and adalimumab can be used in more severe cases [3,5]. Bowel perforation, obstruction, or bleeding are recognized complications of GIBD that necessitate emergent surgery, and the use of immunosuppressive would reduce post-operative complications [3].

The purpose of this report is to highlight the difficulties in establishing the diagnosis and the limited high-quality evidence for managing GI involvement. Moreover, it points out the efficacy of adalimumab biosimilar in complicated presentation.

## Case presentation

This is the case of a 33-year-old male, diagnosed with Behçet’s disease a year prior to presentation to our facility. The disease started years ago with recurrent oral ulcers, followed by bilateral eye pain, photophobia, and blurring of vision treated as blepharitis and conjunctivitis. Over time, his disease progressed, and he developed asymmetrical, migratory, large joint pain and swelling involving the left shoulder, bilateral knees and right wrist and morning stiffness for a year. He was initially diagnosed as seronegative rheumatoid arthritis and managed with prednisolone, methotrexate, and hydroxychloroquine. His joint swelling improved; however, he continued to have recurrent oral aphthous ulcers. As the condition advanced, he developed recurrent genital ulcers followed by recurrent, non-healing lower limb ulcers. He met the clinical diagnostic criteria for Behçet’s disease. Therefore, his treatment was adjusted a month prior to presentation to oral colchicine 0.6 mg twice daily, oral prednisolone 30 mg daily, subcutaneous methotrexate injection 15 mg weekly, oral hydroxychloroquine 200 mg daily in addition to oral azathioprine 50 mg twice daily.

**Figure 1:**
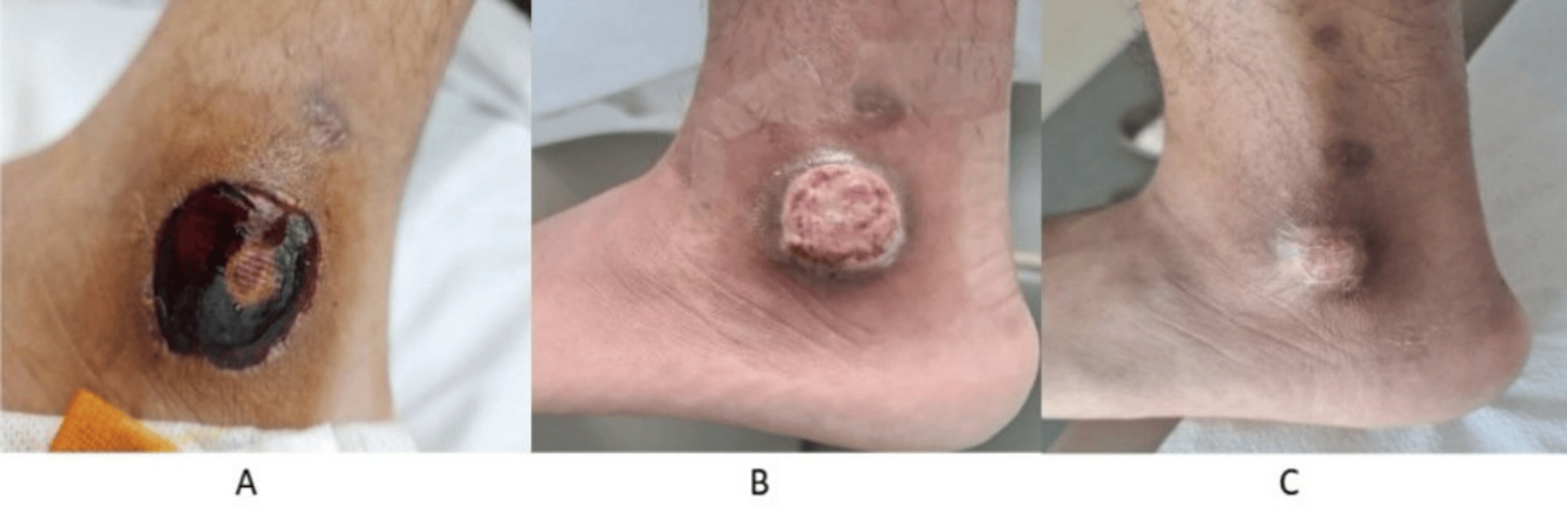
Vasculopathic ulcer pre- and post-treatment. (A) Left lateral peri-malleolar ulcer around 2 cm with underlying collection. (B) After two months of starting anti-TNF-α. (C) Completely healed skin ulcer five months after starting anti-TNF-α. TNF: tumor necrosis factor.

**Figure 2:**
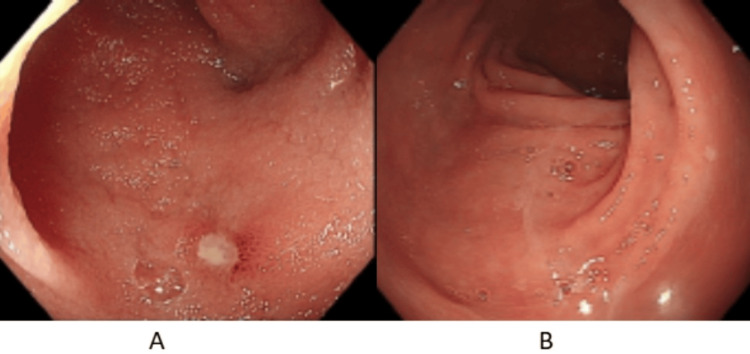
(A) Aphthous ulcer in terminal ileum mucosa on initial colonoscopy. (B) Normal terminal ileum mucosa on repeat colonoscopy eight months after starting anti-TNF-α therapy. TNF: tumor necrosis factor.

**Figure 3:**
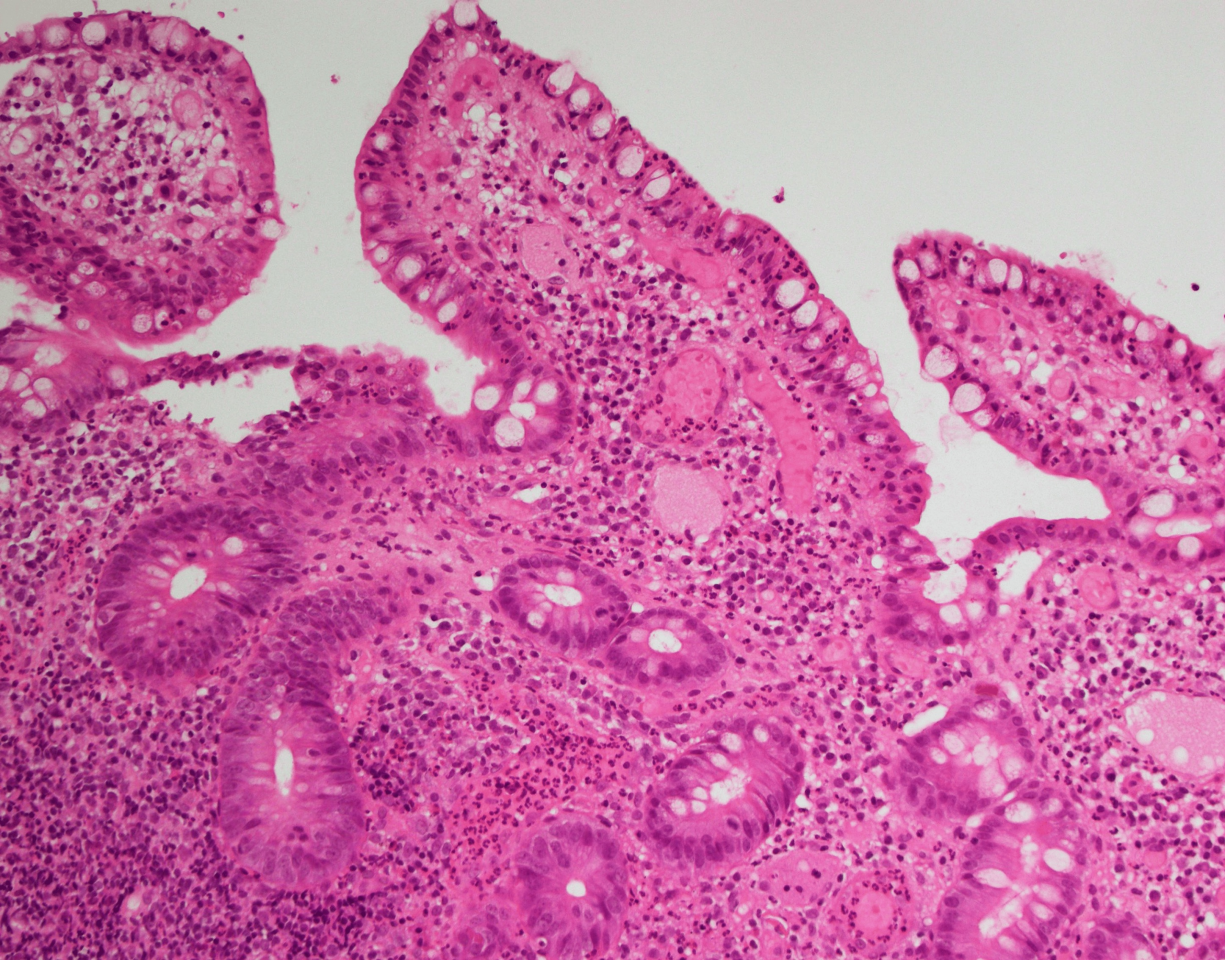
Histopathology image of ileal mucosal ulcer biopsy. Hematoxylin and eosin: Stained biopsy showing distorted, blunted, inflamed, eroded ileal mucosa villi with surface erosion, plasmacytosis, and congested edematous lamina propria with neutrophils.

He presented with two painful, non-healing vasculopathic ulcers over the left mid-leg, and left peri-malleolar region (Figure 1). Each was around 2 cm in size. He reported that his bowel habit has been alternating between episodes of non-bloody diarrhea and constipation for one year, associated with cramping abdominal pain. Laboratory tests revealed elevated markers of inflammation C-reactive protein 54 (<5.0 mg/L), procalcitonin 0.03 (<0.05 ng/mL), erythrocyte sedimentation rate 45 (<11 mm/1 h), and a normal white cell count. Negative anti-neutrophilic cytoplasmic antibody (ANCA), anti-nuclear antibody (ANA), rheumatoid factor, and anti-cyclic citrullinated peptide antibodies (anti-CCP) (Table 1). He was initially treated with IV hydrocortisone 50 mg thrice daily, azathioprine 50 mg twice daily, and broad-spectrum IV antibiotics. He underwent incision and drainage for the collection underneath the malleolar ulcer. He was considered an inadequate responder to conventional disease-modifying anti-rheumatic drugs (CDMARDs), including methotrexate, azathioprine, and hydroxychloroquine. Hence, they were discontinued, and treatment was escalated to adalimumab biosimilar 40 mg subcutaneously every fourteen days. He underwent esophagogastroduodenoscopy (EGD) with biopsy, which revealed mild esophagitis, and a normal biopsy result. Colonoscopy identified a linear ulcer in the ileocecal valve and an aphthous-like ulcer in the terminal ileum (Figure 2). Biopsy of the ulcers showed moderate chronic ileitis with surface erosion and mucosal ulceration, without evidence of granulomas or malignancy (Figure 3). He achieved a complete therapeutic response to adalimumab biosimilar with the healing of vasculopathic skin ulcers, resolution of systemic symptoms, and histopathologic remission of mucosal gastrointestinal ulcers.

**Table 1:** Laboratory investigations. ALT: alanine transaminase; ANA: anti-nuclear antibody; C-ANCA: cytoplasmic anti-neutrophil cytoplasmic antibodies; P-ANCA: perinuclear anti-neutrophil cytoplasmic antibodies; ESR: erythrocyte sedimentation rate; eGFR: estimated glomerular filtration rate; RNP: ribonucleoprotein.

Parameters	Patient values	Reference range
Hemoglobin	11.5	13-17 g/dL
WBC count	9.3 x 10^3^	3.6-11.0 x 10^3^/uL
Platelets count	432 x 10^3^	150-410 x 10^3^/uL
Creatinine	0.78	0.70-1.20 mg/dl
eGFR	120.8	>60 ml/min/1.73 m^2^
Sodium	141	136-145 mmol/L
Potassium	4.0	3.3-4.8 mmol/L
Bicarbonate	28.7	20-28 mmol/L
C-reactive protein	54.4	<5.0 mg/L
ESR	45	<11 mm/1 h
Procalcitonin	0.02	<0.05 ng/mL
Bilirubin	0.36	0-1.0 mg/dl
Alkaline phosphatase	62	40-129 U/L
ALT	9	0-41 U/L
Albumin	4.4	3.4-4.8 g/dL
Anti-RNP-Smith	<0.1	<0.1
Anti-Smith	<0.1	<0.1
Anti-SS-A	<0.1	<0.1
Anti-SS-B	<0.1	<0.1
Anti-scleroderma 70	<0.1	<0.1
Anti-JO-1	<0.1	<0.1
Anti-centromere	<0.1	<0.1
ANA	<1/100	<1/100
Anti-CCP	<8.00	<17 U/mL
C-ANCA	<2.3	<20 CU
P-ANCA	<3.2	<20 CU
Rheumatoid factor	<10	<14 IU/mL

## Discussion

The etiology of BD is currently unknown. It is believed to be multifactorial, including genetic components, immune dysregulation, vascular changes, and hypercoagulability [6]. Alterations in cellular signaling pathways have been linked to Behçet’s disease [6]. Diagnosis is based on clinical criteria, including the International Criteria for Behçet’s disease (ICBD) revised in 2014, which is more sensitive than the International Study Group (ISG) diagnostic criteria for Behçet’s disease created in 1990 [6,7].

GIBD is rarely encountered, and the most frequently reported symptoms include nausea, vomiting, abdominal pain, diarrhea, and gastrointestinal bleeding [8,9]. Common endoscopic findings include mural ulcerations with a polypoid mucosal surface, terminal ileum, and ileocecal valve involvement [6]. The intestinal form of Behcet's disease is confirmed in the presence of typical endoscopic findings. Combined with other symptoms of BD such as recurrent ulcers, oral, and ocular findings [6].

This case report describes a young man with a chronic disease course, which was first misdiagnosed as rheumatoid arthritis due to inflammatory arthritis. As his disease progressed, he developed recurrent genital and vasculopathic ulcers, and subsequently, he met the diagnostic criteria for Behcet’s disease. In addition, he had GI manifestation, which was further investigated by colonoscopy, which showed a linear ulcer in the ileocecal valve and an aphthous-like ulcer in the terminal ileum. Histopathological examination of the ulcers showed moderate chronic ileitis with surface erosion and mucosal ulceration and disease mimickers were ruled out. He failed to respond to CDMARDs and achieved clinical, biochemical, and endoscopic remission after treatment with adalimumab biosimilar.

The management of GIBD is complex due to a lack of standardized guidelines and high-level evidence to support the use of medical therapies [3]. Even though there is encouraging evidence that anti-TNF-α treatment is effective, a prospective, randomized, placebo-controlled study is required to validate their usage [3]. Our study correlates between clinical and endoscopic remission in a patient with gastrointestinal manifestations of Behcet’s disease. 

According to the most recently published British guidelines in 2024, treatment of GIBD should rely on clinical improvement of symptoms in addition to macroscopic and microscopic healing of ulcers on repeated endoscopy to identify patients who require escalation of medical therapy [10]. With regard to regular surveillance, there are no current established guidelines highlighting the need for further research in this area [6].

The initial treatment of mucocutaneous manifestations in BD, according to European Alliance of Associations for Rheumatology (EULAR) guidelines, is colchicine [3]. However, despite the use of colchicine and azathioprine, our patient continued to develop recurrent non-healing ulcers, which eventually responded to anti-TNF-α.

## Conclusions

Behçet's disease is a rare and complex multisystem disorder. Its diagnosis is often delayed due to the gradual emergence of criteria-based clinical features. Increasing evidence supports the use of anti-TNF-α agents in the management of refractory vasculopathic and gastrointestinal Behçet's disease. More robust studies, including randomized controlled trials, are essential to standardize the treatment. Furthermore, this case demonstrates the effectiveness of adalimumab biosimilar in treating refractory Behçet’s disease. 
